# Reassessment of Prostate Biopsy Specimens for Patients Referred for Robot-assisted Radical Prostatectomy Rarely Influences Surgical Planning

**DOI:** 10.1016/j.euros.2021.04.003

**Published:** 2021-04-27

**Authors:** Robert J. Hoekstra, Ward J.H. Goossens, Alexander Beulens, Hilde van Herk, Brigiet M. Hoevenaars, Joost de Baaij, Diederik M. Somford, J.P. Michiel Sedelaar, Jean-Paul A. van Basten, H.J. Eric J. Vrijhof

**Affiliations:** a Department of Urology, Catharina Hospital Eindhoven, Eindhoven, The Netherlands; b Department of Urology, Canisius Wilhelmina Hospital, Nijmegen, The Netherlands; c Prosper Prostate Clinic, Nijmegen, The Netherlands; d Department of Pathology, PAMM Foundation Laboratory for Pathology and Medical Microbiology, Veldhoven, The Netherlands; e Department of Pathology, Canisius Wilhelmina Hospital, Nijmegen, The Netherlands; f Department of Urology, Radboud University Medical Center, Nijmegen, The Netherlands

**Keywords:** Nerve-sparing, Pelvic lymph node dissection, Prostate biopsies, Robot-assisted radical prostatectomy, Surgical planning

## Abstract

**Background:**

The minimum volume standard is 100 robot-assisted radical prostatectomy (RARP) procedures per hospital in the Netherlands, so patients have to be referred to high-volume surgical centers for RARP. During preoperative work-up, prostate biopsies taken elsewhere are reassessed, with upgrading or downgrading of the initial Gleason grade group a possible consequence.

**Objective:**

To determine if prostate biopsy reassessment leads to adjustment of the surgical plan regarding a nerve-sparing approach and extended pelvic lymph node dissection (ePLND) during RARP.

**Design, setting, and participants:**

For 125 men who were referred to the Prosper prostate center at Canisius Wilhelmina Hospital (CWH) in the Netherlands between 2013 and 2016, results for the initial assessment of prostate biopsy by a local uropathologist were compared to results for biopsy reassessment by dedicated uropathologists at CWH.

**Results and limitations:**

The pathologists reached agreement in 80% of the cases. In cases for which there was disagreement (*n* = 25), biopsy revision involved upgrading of the initial grade group in 68% and downgrading in 32%. Biopsy reassessment led to a change in surgical plan in ten cases (8%). As a result of upgrading, ePLND was performed in three patients (2%). ePLND was omitted in one patient (1%) because of downgrading. For three patients (2%) a non–nerve-sparing procedure was planned after upgrading of the initial grade group. For four patients (3%), a unilateral nerve-sparing procedure was performed after downgrading.

**Conclusions:**

This study shows that there is large interobserver agreement between uropathologists in the assessment of Gleason grade group in prostate biopsy specimens. Reassessment rarely leads to a change in surgical plan regarding the indication for a nerve-sparing approach and ePLND. Therefore, reassessment of prostate biopsy before radical prostatectomy can be omitted when the initial pathological assessment was performed by a dedicated uropathologist.

**Patient summary:**

Reassessment of the initial prostate biopsy specimen for patients referred to a specialist center for robot-assisted removal of the prostate rarely influences surgical planning and can be omitted.

## Take Home Message

Reassessment of prostate biopsy specimens taken in another hospital rarely leads to a change in surgical plan regarding the indication for a nerve-sparing approach and extended pelvic lymph node dissection. Therefore, reassessment of prostate biopsy specimens before radical prostatectomy can be omitted.

## 1 Introduction

In the Netherlands, 2413 radical prostatectomies (RPs) were performed in 2017, of which 86% were robot-assisted RP (RARP) [1]. RARP is a complex operation associated with serious side effects [2], [3] such as urinary incontinence, which occurs in 4–26% of patients [4], [5], and erectile dysfunction, occurring in 14–90% [6]. Urinary incontinence and erectile dysfunction are often caused by dissection of the neurovascular bundles for oncological safety [7]. Nerve-sparing surgery (NSS) can only be performed safely in selected patients because negative surgical margins are always the main objective [8]. Patients with high-grade prostate cancer (PCa), defined as Gleason grade group 4 or 5, have a considerable risk of extraprostatic tumor growth and therefore NSS is not recommended by the European Association of Urology for these cases [9]. Moreover, these patients have a higher risk of lymph node invasion, so extended pelvic lymph node dissection (ePLND) is required to assess lymph node status [9], [10]. Since ePLND can lead to serious complications, proper indication for this procedure is mandatory [11].

Surgical experience in RARP is strongly associated with negative surgical margins and preservation of erectile function and urinary continence [12], [13]. Analysis of health insurance claims for reimbursement for pad materials from patients undergoing RARP revealed fewer claims among men operated in hospitals where more than 100 RARP surgeries are performed annually [14]. On the basis of these data, the Dutch Society of Urology increased the minimal annual number of RARP procedures per hospital to 100 in January 2019. To meet the volume standard of 100 procedures, networks and partnerships between hospitals have been formed in which RARP procedures are concentrated at one location [15]. One of the challenges for these collaborations is the uniformity of the diagnostic workup and the indication for RARP.

The Gleason grade group and the number of positive cores determine the indication for and nature of RARP, but histopathological assessment of prostate biopsy specimens is vulnerable to interobserver variability. Reassessment can lead to upgrading or downgrading of grade group and therefore a change in treatment plan [16]. For example, this can change the estimated risk of the presence of lymph node invasion or extraprostatic extension (EPE) according to current nomograms and therefore could have consequences regarding preservation of the neurovascular bundles and the indication for ePLND. Although previous studies have evaluated the results of histopathological reassessment of prostate biopsy specimens [17], [18], it remains unclear to what extent reassessment has consequences for the RARP indications and execution.

In this study, we investigated the prevalence of disagreement between uropathologists after reassessment of biopsy cores, the variables predicting disagreement, and the extent to which reassessment leads to a different indication and RARP execution regarding ePLND and NSS.

## 2 Patients and methods

In 2013, two hospitals (Catharina Hospital Eindhoven, Eindhoven, The Netherlands [CHE] and Canisius Wilhelmina Hospital, Nijmegen, The Netherlands [CWH]) decided to collaborate and concentrate their RARP procedures in a single location (CWH) [15]. Between March 2013 and July 2016, 125 men with PCa diagnosed in CHE who were eligible for RP were referred to CWH.

RARP was performed using a da Vinci Si or Xi robotic platform. Initial diagnostic evaluation was performed in CHE with transrectal ultrasound–guided prostate biopsies and local staging via 3-T multiparametric magnetic resonance imaging (mpMRI). The Gleason grade group was determined according to the International Society of Urological Pathology grading scheme [19], [20]. To determine the risk of lymph node invasion, the Memorial Sloan Kettering Cancer Center (MSKCC) preoperative nomogram was used [21]. In 2013 and 2014, a cutoff value of 10% for the probability of lymph node metastases was used to determine the indication for ePLND, in compliance with the Dutch Prostate Cancer Guideline, whereas from 2015 the cutoff value was lowered to 5% in accordance with the European Association of Urology (EAU) PCa guidelines [9]. On the basis of the initial diagnostic evaluation, patients were referred to CWH for RARP with a surgical plan regarding preservation of the neurovascular bundles and performance of ePLND.

Pathologists in both hospitals are well trained and have more than 30 yr of experience in the diagnosis of PCa. Initial prostate biopsy specimens taken at CHE were initially assessed by pathologists at CHE who specialize in uropathology. Reassessment is performed by two uropathologists in CWH. Depending on the biopsy reassessment results, the surgical plan could be changed, assuming that the biopsy pathology results reassessed at CWH were more accurate.

Every week, all referred patients were reviewed by a team of four urologists. According to the clinical features, reassessed pathology, and evaluation of MRI results, a definitive plan was made regarding the indication for NSS and ePLND. Subsequently, RARP specimens were analyzed by two dedicated uropathologists in CWH and the definitive grade group was compared to the initial grade group assigned on biopsy.

### 2.1 Data analysis

Preoperative, perioperative, and postoperative data were reviewed retrospectively. SPSS v24 was used for the analyses. Comparisons were carried out using χ^2^ and Fisher’s exact test, as appropriate. Nonparametric tests were used to compare the difference in scores by group (for significant differences). Confidence intervals (CIs) for the rate of biopsy agreement after reassessment were calculated using the modified Wald method. Interobserver agreement was calculated using Cohen’s κ, with results interpreted using the guidelines of Landis and Koch for univariate and multivariate analyses [22], [23]. Univariate logistic regression analysis was used to identify factors that could predict agreement of biopsy pathology results. The α level was set at 0.05.

## 3 Results

### 3.1 Demographics

**Fig. 1:**
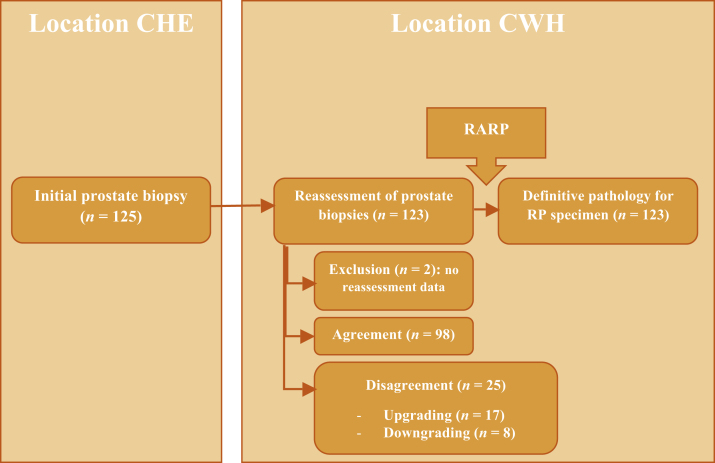
Flow diagram for pathology assessment. CHE = Catharina Hospital Eindhoven; CWH = Canisius Wilhelmina Hospital; RARP = robot-assisted radical prostatectomy.

Of the initial 125 men, two were excluded because of lack of reassessment results, leaving 123 patients eligible for the study (Fig. 1).

The patient characteristics are presented in Table 1. Pathology results for the initial and reassessed biopsy samples and for the RARP specimens are shown in Supplementary Table 1.

**Table 1:** Patient and clinical characteristics (*n* = 123)

Parameter	Result
Median age, yr (range)	65.0 (52.0–74.0)
Median prostate-specific antigen, ng/ml (range)	8.2 (2.5–42.0)
Clinical stage on digital rectal examination, *n* (%)	
cT1c	78 (63.4)
cT2	40 (32.5)
cT3	5 (4.1)
Median prostate volume, ml (range)	40.0 (20.0–150.0)
Median number of cores, *n* (range)	11 (3–18)
Median number of positive cores, *n* (range)	4 (1–13)
Median positive core rate, % (range)	41.67 (6.67–100.00)
PI-RADS score on magnetic resonance imaging, *n* (%)	
PI-RADS 2	4 (3.3)
PI-RADS 3	16 (13.0)
PI-RADS 4	34 (27.6)
PI-RADS 5	32 (26.0)
Unknown	37 (30.0)
Clinical stage on magnetic resonance imaging, *n* (%)	
T0	6 (4.9)
T2	10 (8.1)
T2a	32 (26)
T2b	19 (15.4)
T2c	29 (23.6)
T3a	19 (15.4)
T3b	5 (4.1)
Tx	3 (2.4)

PI-RADS = Prostate Imaging-Reporting and Data System.

### 3.2 Interobserver agreement between initial assessment and biopsy reassessment

The results show a high rate of interobserver agreement for Gleason grade group (79.7%; Table 2) with a κ value of 0.724. The Gleason grade group initially assigned was upgraded in 17 cases and downgraded in eight cases on reassessment.

**Table 2:** Interobserver agreement between the initial Bx assessment and Bx reassessment (*n* = 123)

	Initial Bx pathology vs reassessed Bx pathology
Agreement, *n* (%)	98 (79.7%, 95% CI 71.7–85.9%)
Upgrading, *n* (%) a	17 (13.8%, 95% CI 8.7–21.1%)
Downgrading, *n* (%) b	8 (6.5%, 95% CI 3.1–12.5%)
Cohen’s κ	0.724 (*p* < 0.0001) (substantial level of agreement)

Bx = biopsy; CI = confidence interval calculated using the modified Wald method.

a Upgrading defined as a higher Gleason grade group on Bx reassessment.

b Downgrading defined as a lower Gleason grade group on Bx reassessment.

### 3.3 Variables predicting agreement between initial biopsy assessment and biopsy reassessment

Analysis of the possible factors that could cause disagreement did not identify any significant determinant (Table 3).

**Table 3:** Univariate analysis of factors possibly influencing agreement of pathology results after biopsy revision

	Odds ratio (95% CI)	*p* value
Prostate volume	0.994 (0.974–1.014)	0.562
Initial prostate-specific antigen	0.970 (0.895–1.052)	0.461
Number of cores	0.905 (0.762–1.076)	0.260
Number of positive cores	0.997 (0.852–1.167)	0.970
Percentage of positive cores	1.004 (0.987–1.022)	0.624
Gleason grade group on initial biopsy		
Grade group 1 (reference)	1.000	
Grade group 2	0.358 (0.111–1.140)	0.082
Grade group 3	0.608 (0.145–2.554)	0.497
Grade group 4	1.318 (0.367–4.733)	0.672
Grade group5	0.293 (0.033–2.590)	0.270
Year of biopsy		
2013 (reference)	1.000	
2014	0.386 (0.082–1.815)	0.228
2015	0.584 (0.157–2.172)	0.442
2016	2.640 (0.794–9.305)	0.131
PI-RADS score on magnetic resonance imaging		
PI-RADS 2 (reference)	1.000	
P-IRADS 3	0.692 (0.052–9.210)	0.781
PI-RADS 4	0.932 (0.084–10.154)	0.948
PI-RADS 5	0.840 (0.075–9.384)	0.887
Unknown	0.581 (0.051–6.570)	0.661
Magnetic resonance imaging fusion biopsy performed	1.283 (0.532–3.098)	0.579

CI = confidence interval; PI-RADS = Prostate Imaging-Reporting and Data System.

### 3.4 Changes in surgical strategy due to differences in Gleason grade group between assessment and reassessment

On the basis of the revised biopsy pathology, some changes to the original surgical plan were made regarding NSS and ePLND.

#### 3.4.1 NSS technique and surgical margins

For eight of the 123 patients (6.5%) there was disagreement in biopsy grade group that resulted in a change in surgical management concerning NSS (Table 4). Of the remaining 16 patients for whom there was disagreement in grade group on biopsy reassessment, this difference did not alter the indication for NSS. Among these 16 patients, biopsy reassessment resulted in grade group upgrading in 14, which was confirmed in nine patients on final pathology after RARP.

**Table 4:** Changes in management regarding NS surgery a

Patient	cT stage	GG for initial Bx (CHE)	Planned NS approach	GG for reassessed Bx (CWH)	NS approach	Definitive GG on RP
1	cT2b	GG 2	None	3	UNS right	GG 2
2	cT1c	GG 4	None	2	Bilateral	GG 2
3	cT1c	GG 4	None	1	Bilateral	GG 1
4	cT1c	GG 3	None	2	Bilateral	GG 3
5	cT1c	GG 2	UNS right	1	Bilateral	GG 1
6	cT2b	GG 1	UNS right	2	Non-NS	GG 2
7	cT2x	GG 4	UNS right	5	Non-NS	GG 5
8	cT1c	GG 2	UNS left	3	Non-NS	GG 3

Bx = biopsy; CHE = Catharina Hospital Eindhoven; CWH = Canisius Wilhelmina Hospital; GG = Gleason grade group; NS = nerve-sparing; RP = radical prostatectomy; UNS = unilateral NS.

a In four patients (numbers 1, 6, 7, 8) there was an upgrade in GG on Bx reassessment. Despite the upgrading, the surgical plan for patient 1 changed from non-NS to UNS. In four patients (numbers 2, 3, 4, 5) there was a downgrade in GG on Bx reassessment that was confirmed in three patients. In one patient the definitive pathology was the same as the initial assessment.

#### 3.4.2 ePLND

Among the 25 patients for whom there was disagreement in grade group after biopsy reassessment, the indication for ePLND did not change for 21/123 (17%). Three patients (2%) for whom ePLND was initially not planned during the consultation at CHE eventually underwent ePLND at CWH after grade group upstaging. None of these men had lymph node invasion. For one patient downgraded from grade group 4 to grade group 1 after biopsy reassessment, the indication for ePLND no longer applied and grade group 1 was confirmed on final pathology (Table 5).

**Table 5:** Changes in indication for ePLND

Patient	cT stage	GG for initial Bx (CHE)	N1P before Bx reassessment (%)	ePLND planned on referral	GG for reassessed Bx (CWH)	N1P after Bx reassessment (%)	ePLND performed	GG on RP	pN stage
1	cT2	GG 4 (GS 3 + 5)	5	No	GG 5	23	Yes	5	N0
2	cT2	GG 1	3	No	GG 2	6	Yes	1	N0
3	cT2	GG 1	3	No	GG 2	5	Yes	2	N0
4	cT1c	GG 4	11	Yes	GG 1	1	No	1	Nx

Bx = biopsy; CHE = Catharina Hospital Eindhoven; CWH = Canisius Wilhelmina Hospital; ePLND = extended pelvic lymph node dissection; GG = Gleason grade group; GS = Gleason score; N1P = probability of N1 disease according to the Memorial Sloan Kettering Cancer Center nomogram; RP = radical prostatectomy.

### 3.5 Correlation between biopsy pathology and final pathology at RARP

Comparison of the initial and reassessed biopsy results to the final pathology at RARP is presented in Table 6. These results show higher concordance between the revised biopsy assessment and final pathology than between the initial biopsy result and final pathology.

**Table 6:** Pathology agreement (*n* = 123) for the initial Bx assessment and Bx reassessment versus pathology of the RP specimen and corresponding level of interobserver agreement

	Pathology of initial Bx vs RP	Pathology of reassessed Bx vs RP
Agreement, *n* (%)	57 (46.3%, 95% CI 37.8–55.1%)	67 (54.4%, 95% CI 45.7–63.0%)
Upgrading, *n* (%) a	45 (36.6%, 95% CI 28.6–45.4%)	34 (27.6%, 95% CI 20.5–36.2%)
Downgrading, *n* (%) b	21 (17.1%, 95% CI 11.4–24.8%)	22 (17.9%, 95% CI 12.1–25.7%)
Level of agreement	Fair	Fair
Cohen’s κ	0.282 (*p* < 0.0001)	0.362 (*p* < 0.0001)

Bx = biopsy; CI = confidence interval calculated using the modified Wald method; RP = radical prostatectomy.

a Upgrading defined as a higher Gleason grade group for the RP specimen.

b Downgrading defined as a lower Gleason grade group for the RP specimen.

## 4 Discussion

Owing to the minimum volume standard of 100 RARP procedures annually per hospital in the Netherlands, patients with PCa diagnosed elsewhere have to be referred to a high-volume RARP center. During the preoperative workup, prostate biopsies taken elsewhere are usually reassessed, with upgrading or downgrading of the initial Gleason grade group assigned a possible consequence. Here we presented a series of 125 men with PCa diagnosed at CHE who were referred for RARP to CWH. This is the first study to investigate whether prostate biopsy reassessment actually leads to a change in surgical plan in terms of the indication for NSS and ePLND during RARP.

### 4.1 How often does reassessment of biopsy pathology lead to a change in Gleason grade group?

Reassessment of biopsy specimens at CWH resulted in confirmation of the original pathology results from CHE in 79.7% of the patients (Table 2). Almost 14% of the initial pathology results were upgraded to a higher grade group, whereas in 6.5% of the initial results were downgraded. The interobserver agreement between the two results was substantial (κ = 0.724). Univariate analysis did not identify any specific determinant related to disagreement between initial and second assessments of prostate biopsy pathology. Our results show a higher level of agreement compared to the 67% between initial biopsy assessment and internal reassessment reported by Truesdale et al [24]. This may be explained by the fact that biopsies were initially assessed by a dedicated uropathologist, in contrast to most other studies, in which second opinions on prostate biopsy sample from uropathologists are compared to initial assessments by general pathologists in community hospitals [24], [25], [26].

### 4.2 Has reassessment of biopsy pathology led to changes in surgical approach regarding NSS and ePLND?

In the present series, biopsy reassessment resulted in a change to NSS strategy for only eight patients (6.5% of the whole group) and was beneficial in four patients who underwent NSS and for whom non-NSS was otherwise planned (Table 4). All four patients had negative surgical margins. Three patients for whom NSS was planned underwent non-NSS after biopsy reassessment; all three patients had EPE and one of them unfortunately had a positive surgical margin. Because NSS is contraindicated if EPE is suspected, it is likely that these men have benefited from the reassessment.

The presence of (side-specific) EPE is the main factor when selecting patients for NSS in RARP. Before the introduction of prostate MRI, (side-specific) EPE was estimated using prediction models such as the MSKCC nomogram. Although MRI technology is improving and the applicability of MRI for PCa staging is increasing, new nomograms for prediction of side-specific EPE have been proposed in recent years. This emphasizes the remaining need for additional tools for adequate selection of patients for NSS, as the sensitivity of mpMRI for EPE generally remains low (57%). Factors other than MRI, such as the number of side-specific positive biopsies and the grade group, are crucial in this respect.

According to EAU PCa guidelines, ePLND should be performed when the risk of lymph node metastasis exceeds 5% according to prevailing nomograms [9], [17], [18], [27]. After biopsy reassessment, only three patients (2% of the whole group) were pushed over the indication level for ePLND (Table 5). These patients underwent ePLND that was not planned initially, but none of the three had lymph node metastases.

Summarizing these results, the surgical plan for RARP is rarely changed after revision of the biopsy pathology. The occasional discordance in prostate biopsy pathology between the two hospitals is just as likely to occur between uropathologists in the same referral clinic. Therefore, our idea is to terminate reassessment of biopsy pathology in cases for which the prostate biopsy was initially assessed by a dedicated uropathologist. Omitting reassessment of biopsy pathology will save Є315.06–346.57 per RARP procedure in the Dutch setting.

### 4.3 How do the original and revised histopathology results for biopsy cores correlate to the definitive histopathology results after RP?

When comparing pathology results for the initial biopsy assessment to the definitive pathology at RP, we found agreement of 46.3%. After revision of the biopsy pathology at CWH, the agreement level increased to 54.4%. The interobserver agreement was fair for both comparisons (Table 6).

The reproducibility of PCa pathology is a well-known issue [16], [28]. One consideration is that prostate biopsy grade groups are reported differently to RP grade groups [19], [29]. In addition, possible sampling bias needs to be acknowledged when taking prostate biopsy samples. Current grading systems are still subject to varying individual interpretations, which is reflected in low agreement rates, even for postoperative prostate specimens, for which sampling errors should not be an issue [30].

### 4.4 Limitations

Our study has some limitations. First, this is a retrospective study, with the inherent limitations of this type of study. Second, we were not able to identify all the risk factors besides pathology revision that can influence decision-making regarding the surgical approach for RARP. Finally, this was a two-center study with well-trained pathologists in the referring center. Both are large oncology referral centers with strong affiliations to academic institutions. Therefore, the results are less generalizable to other hospitals.

## 5 Conclusions

In conclusion, pathological reassessment led to a change in biopsy grading in only 20% of cases and rarely (8%) had relevant consequences for surgical planning for robot assisted radical prostatectomy. In this study, initial pathological assessment was performed by a dedicated uropathologist. In our center, a total of 200 prostate biopsy samples are revised yearly. Omitting reassessment of initial prostate biopsy specimens taken elsewhere will save an estimated 120–150 working hours and approximately Є63 000–69 500 per year. Balancing the costs and benefits of re-assessment of prostate biopsies, we terminated reassessment of prostate biopsy samples from patients referred for RARP. Reassessment of biopsy pathology has no added value and can be omitted.

  ***Author contributions***: Michiel J.P. Sedelaar had full access to all the data in the study and takes responsibility for the integrity of the data and the accuracy of the data analysis.

  *Study concept and design*: Goossens, Vrijhof.

*Acquisition of data*: Hoekstra, Goossens, Beulens, Hoevenaars, van Herk, de Baaij.

*Analysis and interpretation of data*: Hoekstra, Goossens, Beulens.

*Drafting of the manuscript*: Hoekstra, Goossens.

*Critical revision of the manuscript for important intellectual content*: Beulens, Somford, Sedelaar, van Basten, Vrijhof.

*Statistical analysis*: Hoekstra, Goossens, Beulens.

*Obtaining funding*: None.

*Administrative, technical, or material support*: Beulens.

*Supervision*: Sedelaar, Basten, Vrijhof.

*Other*: None.

  ***Financial disclosures:*** Michiel J.P. Sedelaar certifies that all conflicts of interest, including specific financial interests and relationships and affiliations relevant to the subject matter or materials discussed in the manuscript (eg, employment/affiliation, grants or funding, consultancies, honoraria, stock ownership or options, expert testimony, royalties, or patents filed, received, or pending), are the following: None.

  ***Funding/Support and role of the sponsor*:** None.

  ***Acknowledgments*:** The authors would like to thank Saskia Houterman for her help with the statistical analysis.

## CRediT authorship contribution statement

**Robert J. Hoekstra:** Validation, Formal analysis, Investigation, Data curation, Writing - original draft, Visualization. **Ward J.H. Goossens:** Conceptualization, Methodology, Software, Formal analysis, Investigation, Data curation, Writing - original draft. **Alexander Beulens:** Methodology, Software, Validation, Formal analysis. **Hilde van Herk:** Investigation, Resources. **Brigiet M. Hoevenaars:** Investigation, Resources. **Joost de Baaij:** Investigation, Resources, Data curation. **Diederik M. Somford:** Writing - review & editing. **Michiel J.P. Sedelaar:** Writing - review & editing, Visualization, Supervision, Project administration. **Jean-Paul A. van Basten:** Validation, Writing - review & editing, Supervision. **H.J. Eric J. Vrijhof:** Conceptualization, Methodology, Validation, Writing - review & editing, Supervision, Project administration.
