# Supplementary material for: Reassessment of Prostate Biopsy Specimens for Patients Referred for Robot-assisted Radical Prostatectomy Rarely Influences Surgical Planning
Source: Eur Urol Open Sci. 2021 Apr 27;28:36–42. doi: 10.1016/j.euros.2021.04.003 (PMC8317876; doi:10.1016/j.euros.2021.04.003)
Supplement: Supplementary file 1 [file mmc1.docx]

**Supplementary Table 1 – Pathology results of the initial biopsies, the reassessment of the biopsies and the definitive pathology of the prostatectomy specimen.**

|  | Initial Biopsy ISUP grades (Hospital A)*, n (%)* | Reassessment Biopsy ISUP grades (Hospital B)*, n (%)* | Prostate specimen ISUP grades (Hospital B)*, n (%)* |
| --- | --- | --- | --- |
| **All patients (n=123)** | | | |
| ISUP grade 1 | 40 (35.3) | 33 (26.8) | 20 (16.3) |
| ISUP grade 2 | 42 (34.1) | 50 (40.6) | 56 (45.5) |
| ISUP grade 3 | 16 (13.1) | 17 (13.8) | 24 (19.5) |
| ISUP grade 4 | 15 (12.2) | 10 (8.1) | 12 (9.7) |
| ISUP grade 5 | 10 (8.1) | 18 (14.6) | 11 (8.9) |
